# Process-Controlled Functional Polymer Films on Paper: Oxygen Barrier and Antimicrobial Performance of PVA–Amylose Coatings

**DOI:** 10.3390/polym18020264

**Published:** 2026-01-19

**Authors:** Korakot Charoensri, Dae Hyeon Kwon, Hong Seok Kim, Intatch Hongrattanavichit, Yang Jai Shin, Hyun Jin Park

**Affiliations:** 1 Department of Imaging and Printing Technology, Faculty of Science, Chulalongkorn University, Bangkok 10330, Thailand; korakot.c@chula.ac.th (K.C.); intatch.h@chula.ac.th (I.H.); 2 Department of Biotechnology, College of Life Sciences and Biotechnology, Korea University, 145 Anam-ro, Seongbuk-gu, Seoul 02841, Republic of Korea; dhkwon@youlchon.com; 3 Research and Development Center, Youlchon Chemical, Co., Ltd., Beomjigi-ro, Danwon-gu, Ansan-si 425-851, Gyeonggi-do, Republic of Korea; hongseok0895@youlchon.com

**Keywords:** PVA, amylose, ZnO oxide nanoparticles, oxygen barrier coating, paper-based packaging

## Abstract

The development of functional polymer films on porous paper substrates is inherently constrained by substrate-induced defects that hinder film continuity and barrier performance. In this study, process-controlled amylose–Poly(Vinyl alcohol) (PVA) coatings incorporating ZnO nanoparticles (ZnO NPs) were fabricated via aqueous deposition to investigate the process-structure-property relationship governing oxygen barrier behavior on paper. The moisture resistance of the coating was also evaluated. Single-layer coatings exhibited severe barrier failure due to insufficient film formation and pervasive pinhole defects. In contrast, systematic multi-layer deposition enabled the formation of continuous polymer films. A pronounced non-linear reduction in oxygen transmission rate was observed once the dry coating thickness exceeded approximately 5 µm. Under these conditions, the oxygen transmission rate decreased to approximately 15 cc/m^2^·day·atm at 20 °C and 65% relative humidity. This transition was correlated with the elimination of substrate-induced defects, as confirmed by morphological analysis. In addition to enhanced barrier performance, ZnO NP-loaded coatings demonstrated strong and broad-spectrum antimicrobial activity against both *Escherichia coli* and *Staphylococcus aureus*, indicating their multifunctional potential for active packaging applications. Supporting evaluations further indicated adequate mechanical flexibility and high repulpability, highlighting the suitability of the coating for sustainable paper-based packaging. Overall, this work identifies a quantitative critical film thickness that serves as process-specific design guideline for engineering high-performance functional polymer coatings on porous paper substrates.

## 1. Introduction

Plastic packaging waste has become a global environmental challenge, driving an urgent search for sustainable and recyclable alternatives. Governments worldwide are enacting stricter regulations on single-use plastics and mandating a greater use of recyclable or compostable materials. This regulatory pressure—combined with growing consumer demand for eco-friendly packaging—has accelerated interest in alternatives that maintain the protective functions needed for food safety and shelf life [1,2,3,4]. Fiber-based materials, particularly paper and paperboard, are gaining popularity as packaging substrates due to their renewable origin, biodegradability, and established recycling infrastructure. However, uncoated paper lacks sufficient barrier properties against oxygen and moisture, which limits its use for perishable or oxygen-sensitive food products [5,6,7]. Enhancing paper with functional coatings that deliver high barrier performance while maintaining recyclability is therefore a key focus in the development of next-generation sustainable packaging. Biodegradable polyesters such as polylactic acid (PLA), poly(butylene adipate-co-terephthalate) (PBAT), and poly(butylene succinate) (PBS) have been widely explored for paper coating applications. These materials offer mechanical robustness and biodegradability, yet they exhibit several shortcomings. Their moderate oxygen barrier performance often falls short of food packaging requirements, and their hydrophobic nature interferes with fiber recovery during recycling [8,9,10,11,12]. Moreover, the high cost of these polymers poses economic barriers to widespread adoption, especially for high-volume packaging applications. Water-soluble biopolymers have emerged as promising alternatives for functional paper coatings, particularly PVA and starch [13,14]. PVA is a synthetic, yet biodegradable, polymer known for its excellent film-forming ability and outstanding oxygen barrier under dry conditions. Its water solubility allows for efficient repulping during recycling, preserving the core sustainability advantage of paper packaging. However, PVA suffers from moisture sensitivity, elevated production costs, and relatively slow biodegradation under certain composting conditions [15,16,17,18,19]. Amylose, the linear fraction of starch, offers complementary attributes. It is derived from abundant agricultural sources and is cost-effective, biodegradable, and capable of forming dense hydrogen-bonded networks with low gas permeability [20,21,22]. High-amylose starches, in particular, yield films with improved mechanical and barrier properties compared to amylopectin-rich starches. When combined with PVA, amylose enhances biodegradability and reduces the formulation cost, while the resulting blends exhibit compatible hydrogen bonding and good film uniformity. Importantly, both components retain water solubility, supporting recyclability through conventional pulping processes. To further improve the performance of bio-based coatings, functional nanoparticles have been introduced into polymer matrices. ZnO NPs have attracted considerable attention due to their multifunctional properties. Their inclusion improves barrier performance by increasing diffusion path tortuosity, reducing polymer free volume, and promoting matrix densification [23,24]. Additionally, ZnO acts as a nucleating agent, enhancing crystallinity in semi-crystalline polymers like PVA. These effects contribute to significant reductions in both oxygen and water vapor transmission at low ZnO loadings. Beyond passive barrier enhancement, ZnO provides active antimicrobial and UV-blocking functionalities. Its ability to generate reactive oxygen species and release Zn^2+^ ions disrupts microbial cells, offering broad-spectrum antimicrobial protection [24,25]. This property is particularly valuable for packaging perishable food products prone to microbial spoilage. ZnO also exhibits strong UV absorption in the UVA and UVB regions, shielding light-sensitive products from photo-degradation without compromising visual clarity of the packaging [25,26].While material selection is central to functional performance, the application of coatings onto porous substrates such as paper introduces significant technical challenges. Paper surfaces are characterized by high roughness and porosity, leading to partial absorption of water-based coatings. This absorption reduces surface coverage and often results in incomplete films with pinholes and structural defects. Such defects act as direct pathways for gas permeation, nullifying the potential barrier of otherwise effective materials. A key parameter in coating design is the critical film thickness, the minimum thickness at which a coating forms a continuous, defect-free layer over the substrate. Below this threshold, permeation is dominated by film discontinuities; above it, the measured barrier reflects the intrinsic material properties [24,27,28]. For paper substrates, this critical thickness generally falls within the 3–10 µm range, depending on surface morphology and coating formulation. Achieving uniform coverage at or above this threshold, without excessive material use, requires optimization of the coating process, including deposition technique, number of passes, and drying conditions [29,30,31].

This study focuses on the integration of an amylose–PVA biopolymer system reinforced with ZnO nanoparticles into paper substrates for food packaging applications. The primary objective is to determine the critical coating thickness required to form a continuous, defect-free film and to establish the relationship between coating process parameters, film structure, and functional performance. Barrier properties are evaluated based on oxygen and water vapor transmission rates, while antimicrobial efficacy is assessed against common foodborne pathogens. Mechanical robustness is characterized by folding endurance testing. The novelty of this work lies in its systematic investigation of the process–structure–property relationship for water-soluble nanocomposite coatings applied to porous paper substrates. Emphasis is placed not only on material formulation but also on the optimization of processing parameters critical to achieving high-performance barrier coatings.

## 2. Materials and Methods

### 2.1. Materials

PVA and amylose were used as the polymer matrix components. PVA (≈99% hydrolyzed, average MW ~85,000 g/mol) and amylose (from maize, analytical grade; molecular weight not specified by the supplier due to its polydisperse nature) were both obtained from Sigma-Aldrich (St. Louis, MO, USA) and were used as received. ZnO NPs were synthesized in-house following a precipitation method as described in our previous work [25,26]. The base substrate was a pre-sized paper (Neopore FLEX, 60 g/m^2^) characterized by a low water absorptiveness (Cobb 60 g/m^2^ ≤ 20 g/m^2^ for 60 s) to minimize water uptake. All other chemicals (solvents and reagents) were of analytical grade. Deionized (DI) water was used for all solution preparations.

### 2.2. Preparation of PVA–Amylose–ZnO Coating Solution

A homogenous PVA–amylose/ZnO coating solution was prepared by a two-step process. First, PVA was dissolved in hot water: PVA powder (5 g) was added to 100 mL of DI water and heated to 85–90 °C on a magnetic stirrer hotplate. The mixture was continuously stirred at 300 rpm for 30 min until the PVA completely dissolved, yielding a clear viscous solution. Separately, amylose (5 g) and ZnO nanoparticles (0.5 g, 5 wt% of total solids in the reference formulation) were dispersed in 50 mL of DI water. The dispersion was subjected to ultrasonication using an ultrasonic processor (UP200St, Hielscher GmbH, Germany; 200 W, 26 kHz) for 10 min to deagglomerate the ZnO and obtain a uniform nanoparticle dispersion. After sonication, the amylose–ZnO premix was slowly added to the hot PVA solution under continuous stirring. The combined mixture was maintained at 85 °C and was stirred for an additional 30 min to ensure complete dissolution of amylose and uniform distribution of ZnO nanoparticles. The resulting PVA–amylose/ZnO coating solution has a total solid content of approximately 10% (*w*/*v*). The pH of the solution was approximately neutral (pH ~7) and was not adjusted. Prior to coating, the solution was cooled to room temperature and was degassed under mild vacuum to remove any entrained air bubbles, yielding a smooth, homogeneous coating formulation ready for application. For antimicrobial evaluation, additional coating formulations containing lower ZnO nanoparticle loadings (0.5 and 1 wt% relative to total polymer solids) were prepared as described in Section 2.8 to assess concentration-dependent antibacterial performance.

### 2.3. Coating Application and Process Control

The PVA–amylose/ZnO coating was applied using a wire-wound bar coater (RDS #4, R.D. Specialties Inc., Webster, NY, USA) under controlled laboratory conditions (25 °C, 50% RH). Each substrate was affixed to a vacuum platen, and the coating was deposited in a single pass, yielding a wet film thickness of ~100µm. Drying was conducted in a hot-air oven at 60 °C for 10min, followed by ambient air-drying at 25 °C for 24h. Coating add-on was calculated gravimetrically from the mass difference before and after coating (samples were conditioned at 23 °C, 50% RH). Coating thickness was measured using a digital micrometer (Mitutoyo, Mitoyo, Japan) at five randomly selected locations per sample. All coated specimens were stored in a desiccator (25 °C, 50% RH) for ≥48h prior to testing.

### 2.4. Scanning Electron Microscopy (SEM)

Surface and cross-sectional morphology of coated papers was examined using SEM (S-3400N, Hitachi High-Tech, Tokyo, Japan) at 15kV accelerating voltage. Samples were cryo-fractured in liquid nitrogen and sputter-coated with ~5nm platinum. Micrographs were acquired at a range of magnifications to evaluate film continuity, thickness, and nanoparticle dispersion.

### 2.5. KIT Test

Coating continuity and liquid resistance were evaluated using the TAPPI KIT test (TAPPI T559), where ratings from 1 to 12 indicated increasing resistance to liquid penetration, with higher values corresponding to more continuous and defect-free coatings.

### 2.6. Oxygen Transmission Rate (OTR)

The oxygen barrier property was characterized by OTR measurements using a MOCON OX-TRAN 2/21 oxygen permeation analyzer (AMETEK MOCON, Minneapolis, MN, USA). Tests were performed following ASTM D3985, the standard coulometric method for oxygen transmission through films. Coated paper samples were mounted in diffusion cells, separating pure oxygen (99.9% O_2_) on one side and nitrogen (carrier gas) on the other. The test temperature and humidity were set to 23 °C and 0% RH (dry carrier gas), unless stated otherwise. Additional measurements at 20 °C and 65% RH were performed to evaluate humidity-relevant barrier performance. Oxygen that permeates through the sample into the nitrogen side is detected by a coulometric sensor, and the steady-state transmission rate is measured. OTR values (reported in cc·m^−2^·day^−1^ at 1 atm O_2_ partial pressure) were obtained for at least three specimens per sample type. The base paper’s OTR (which is typically high due to the paper’s porous structure) was contrasted with that of the coated paper. The reduction in OTR after coating quantifies the enhancement in oxygen barrier due to the PVA–amylose/ZnO layer.

### 2.7. Water Absorptiveness (Cobb Test)

To assess the surface water resistance imparted by the coating, Cobb tests were performed according to ISO 535:2014 (Cobb60 method). In this test, a known area of the sample (100 cm^2^) is exposed to distilled water for a fixed time (60 s), after which the excess water is removed and the mass of water absorbed is determined. A Cobb tester with a 100 cm^2^ brass ring was used. The initial dry weight of each paper sample was recorded, then 100 mL of water was poured into the test ring placed on the sample. After 60 s contact, the water was, drained and the sample surface was blotted with standardized blotting paper. The sample was weighed again immediately, and the Cobb60 value (g/m^2^) was calculated as the mass of water absorbed per unit area. Both uncoated and coated paper samples were tested (five replicates each).

### 2.8. Antibacterial Activity Testing

The functional bioactive property imparted by ZnO nanoparticles was evaluated by antibacterial testing against two representative foodborne bacteria: *Escherichia coli* (Gram-negative) and *Staphylococcus aureus* (Gram-positive). Coated paper samples containing ZnO nanoparticle loadings of 0.5 and 1 wt% (relative to total polymer solids) were evaluated to examine the concentration-dependent antibacterial activity of the coating system. The antibacterial efficacy of the coated paper was measured following a modified JIS Z 2801:2020 method. Briefly, coated paper samples (50 mm × 50 mm) and control uncoated paper samples were sterilized under UV light and placed in sterile Petri dishes. An inoculum of ~10^5^ colony-forming units (CFU) of each test bacterium (prepared in nutrient broth) was spread uniformly onto the sample surface (under a sterile cover film to ensure full contact). The inoculated samples were then incubated at 37 °C for 24 h at ~95% relative humidity. After incubation, the bacteria were recovered from each sample by vortexing in neutralizing buffer, and the surviving viable cells were quantified by plating and counting colonies. The antibacterial activity was expressed in terms of the log_10_ reduction in CFU on the coated sample compared to the uncoated control. In addition, qualitative zone-of-inhibition tests were performed by placing coated paper disks on agar plates inoculated with the bacteria and observing any clear inhibition halos after 24 h at 37 °C.

### 2.9. Statistical Analysis

Statistical analyses were performed using ANOVA with SPSS software (SPSS 25, IBM, Chicago, IL, USA). Duncan’s multiple range test was used to indicate the statistical differences among the mean values. The results were considered statistically significant at *p* < 0.05. Data are presented as the mean ± standard deviation for each experiment.

## 3. Results and Discussion

### 3.1. Catastrophic Barrier Failure and the Critical Role of Structural Continuity

**Figure 1:**
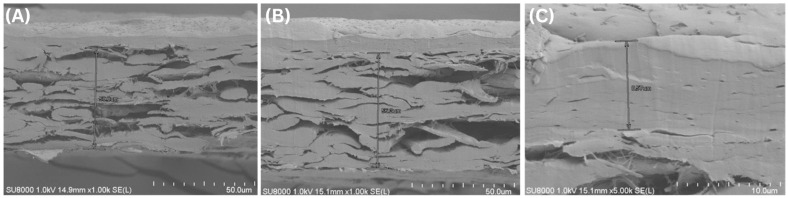
Cross-sectional SEM images of paper substrates coated with amylose–PVA composite films prepared using different coating strategies. (**A**) Single-layer coating showing extensive polymer penetration into the porous paper structure and the absence of a continuous surface film. (**B**) Multi-layer coating (low magnification) illustrating progressive film buildup on the paper surface. (**C**) Multi-layer coating (high magnification) revealing a uniform and continuous coating layer with a well-defined thickness and clear coating–substrate interface. Scale bars: 50 μm (**A**,**B**) and 10 μm (**C**).

**Figure 2:**
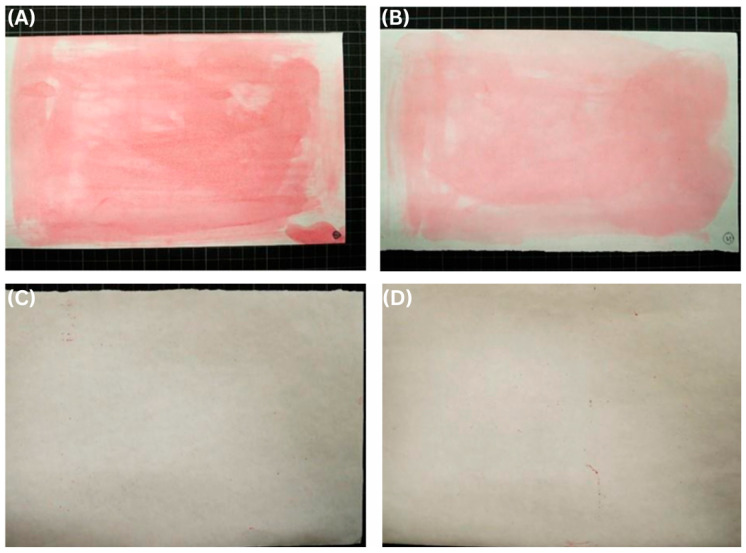
Representative KIT test images illustrating coating continuity on paper substrates. (**A**,**B**) Uncoated paper showing extensive dye penetration due to surface defects and pinholes. (**C**,**D**) Multi-layer amylose–PVA/ZnO-coated paper exhibiting minimal dye penetration, indicating improved coating continuity.

Initial coating trials employing a single-layer deposition on the PVA-sized paper substrate immediately revealed a fundamental limitation associated with applying aqueous biopolymer coatings onto porous materials. The fibrous and rough surface of paper promoted capillary absorption of the coating solution, inhibiting the formation of a uniform surface film. As a result, the dry coating layer was exceedingly thin (approximately 0.8–1.3 µm). Cross-sectional SEM observations confirmed the presence of widespread pinhole defects that exposed the underlying paper fibers (Figure 1A). These structural deficiencies translated directly into catastrophic barrier failure. Oxygen transmission rate (OTR) measurements showed that all single-layer coated samples exhibited OTR values exceeding 1000 cc/m^2^·day·atm. The result indicates no meaningful improvement compared to the uncoated paper substrate. Moreover, noticeable variability among samples was observed, reflecting the non-uniform and defect-dominated nature of the coating layer. Complementary KIT testing further corroborated this assessment, with low KIT ratings (on the order of 1–3) indicative of poor coating continuity and rapid liquid penetration through pinholes. This contrast is illustrated by representative KIT test images: uncoated paper exhibited extensive dye penetration, whereas multi-layer coated samples showed minimal liquid intrusion (Figure 2).

To address this failure mode, a multi-layer deposition strategy was adopted to increase coating thickness and promote structural continuity across the paper surface. By sequentially applying multiple coating layers with intermediate drying steps, substrate-induced defects were progressively sealed. As a result, a more continuous surface film formed, as clearly observed in the multi-layer coated samples (Figure 1B,C). This process-driven approach successfully resolved the catastrophic failure seen in single-layer coatings and enabled the recovery of functional oxygen barrier performance, as summarized in Table 1. Increasing the number of coating layers from 1× to 6× raised the dry coating thickness from ~1 µm to ~5.4–6.6 µm. This increase coincided with a pronounced reduction in OTR >1000 cc/m^2^·day·atm to ~15 cc/m^2^·day·atm (at 20 °C, 65% RH). The dramatic improvement between 4× and 6× coatings suggests that a critical thickness threshold was surpassed. Once the dry coating thickness exceeded approximately 5 µm, a continuous and defect-free barrier layer was achieved. Consequently, oxygen permeability decreased by nearly two orders of magnitude compared to single-layer coatings. This finding validates the concept of a minimum effective thickness for paper coatings and aligns with literature reports for similar system [28,32].

Figure 1 confirms the morphological basis for this transition. Quantitative thickness measurements obtained from multiple locations across each sample (Table 1) further support this interpretation. In particular, the narrower thickness distribution observed for the 6× coating indicates improved coating homogeneity, which correlates with the elimination of localized thin regions and pinhole defects seen in SEM images. This reduction in thickness variability is consistent with the sharp decrease in OTR observed beyond the critical thickness. Coatings below the critical thickness (e.g., Figure 1A for 1× layer) show discontinuous coverage with polymer deeply penetrated into the paper pores and many voids at the surface. In contrast, at 6× layers (Figure 1C), a uniform and continuous coating layer covers the paper, with a well-defined thickness and a clear interface between the coating and substrate. The multi-layer build-up effectively fills in the surface asperities and pores of the base paper, creating a flat top surface. This observation is consistent with prior work on bar-coated paper substrates, where a ~5 µm dry coating was sufficient to cover the substrate without conforming to its roughness [32,33,34]. The elimination of substrate exposure and pinholes beyond the critical thickness explains the non-linear enhancement in barrier properties.

### 3.2. Identification of a Critical Film Thickness for Effective Barrier Performance

**Figure 3:**
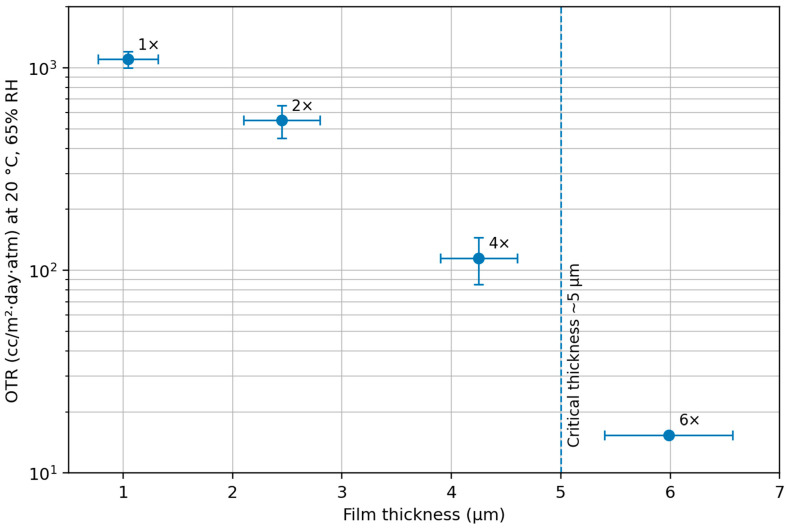
Oxygen transmission rate (OTR) as a function of dry film thickness for PVA–amylose/ZnO coatings on PVA-sized paper measured at 20 °C and 65% RH. The dashed line indicates the critical thickness (~5 µm) required to achieve a continuous oxygen barrier layer.

To quantitatively validate the existence of a critical film thickness governing oxygen barrier performance, the relationship between coating passes, resulting film thickness, and functional barrier properties was systematically examined. By increasing the number of coating layers, the transition from a defect-dominated regime to an effective continuous barrier layer could be clearly resolved. The relationship between oxygen barrier performance and coating thickness is illustrated in Figure 3. The data highlight a pronounced non-linear improvement associated with the formation of a continuous barrier layer. As summarized in Table 1, the bar-coating process exhibited good reproducibility, with each additional coating pass contributing approximately 0.8–1.1 µm to the total dry film thickness. However, reductions in OTR did not scale proportionally with thickness. Coatings below approximately 5 µm, including double- and quadruple-layer systems (~2–4 µm thick), showed only moderate barrier improvement and remained associated with intermediate KIT ratings (indicating incomplete structural continuity). A pronounced non-linear transition in barrier performance was observed once the total coating thickness exceeded ~5 µm. Increasing the number of coating passes from four to six resulted in a thickness increase from 3.9–4.6 µm to 5.4–6.6 µm, accompanied by a sharp decrease in OTR to 15.2–15.6 cc/m^2^·day·atm (20 °C, 65% RH). Notably, the abrupt change in slope of OTR versus coating thickness indicates a non-linear transport regime, supporting a percolation-type transition rather than a purely linear diffusion-controlled scaling. This abrupt improvement identifies ~5 µm as the critical thickness threshold beyond which the coating achieves sufficient continuity to function as an effective oxygen barrier on a porous paper substrate. SEM analysis provided direct morphological confirmation of this transition (Figure 1B,C): coatings below the critical thickness exhibited discontinuous coverage and residual defects, whereas the hexa-layer (~6 µm) coating formed a uniform, continuous film fully sealing the surface roughness and porosity. Cross-sectional images showed a distinct coating layer with thickness consistent with micrometer measurements and good interfacial adhesion to the substrate. Additionally, ZnO nanoparticles were observed to be homogeneously distributed throughout the polymer matrix, indicating stable incorporation without significant agglomeration.

Taken together, these results indicate a transition from a defect-dominated, percolative transport regime to a diffusion-controlled barrier regime once coating continuity is established, consistent with barrier models reported for coated porous substrates. The observed reduction in oxygen permeability is governed by two complementary, process-enabled mechanisms. First, the establishment of a continuous, defect-free coating layer eliminates direct gas transport pathways associated with the porous paper substrate. Second, the incorporation of nano-ZnO increases diffusion path length (tortuosity) and contributes to matrix densification, effects that have been widely reported for PVA- and polysaccharide-based nanocomposites [35,36,37,38]. In this study, both mechanisms are activated once the critical thickness is reached, resulting in an oxygen transmission rate of ~15 cc/m^2^·day·atm for the optimized 6-layer coating. In practical terms, achieving an OTR of approximately 15 cc/m^2^·day·atm on a highly porous paper substrate represents a significant technical milestone. While this value does not yet reach the ultra-high barrier levels characteristic of specialty multilayer films (e.g., PVA or metallized coatings can have OTR < 0.1 cc/m^2^·day) [39,40], it firmly positions the present bio-based coating within the performance range required for many dry food applications. The results demonstrate that by focusing on process-driven structure formation, one can attain excellent barrier performance from inherently water-soluble, bio-derived polymers, without the need for fluoropolymers or other persistent hydrophobic treatments.

### 3.3. Multifunctional Performance: Validation of Active Antimicrobial Functionality

**Figure 4:**
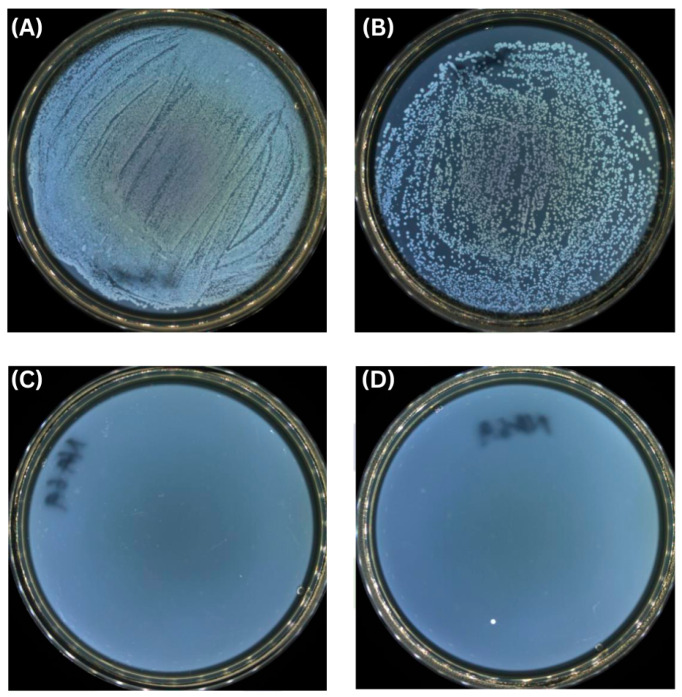
Antimicrobial activity of PVA–amylose/ZnO-coated paper against *Escherichia coli* and *Staphylococcus aureus*. (**A**,**B**) Control samples and (**C**,**D**) coated samples after bacterial incubation.

In addition to achieving effective oxygen barrier performance through process-controlled structural continuity, the incorporation of nano-ZnO imparts active antimicrobial functionality to the coating system, enabling a multifunctional packaging material capable of both passive protection and active spoilage inhibition. Such dual functionality is particularly relevant for extending the shelf life and safety of packaged food products [41,42]. Antimicrobial testing demonstrated that the optimized PVA–amylose/ZnO coating exhibited strong and broad-spectrum antibacterial activity against representative food-borne pathogens. Quantitative viable-count results confirmed a >99.99% reduction in both *E. coli* and *S. aureus* on contact with the coated surface (Table 2). Representative agar plate images further illustrate the near-complete suppression of bacterial colonies on coated samples, in stark contrast to the dense growth observed on uncoated controls (Figure 4). This level of antimicrobial performance indicates rapid and effective bacterial inactivation upon contact with the coating. Similar antimicrobial behavior has been reported previously for ZnO-containing starch-based films [43,44,45,46].

The observed antimicrobial activity is primarily attributed to the presence of ZnO nanoparticles within the polymer matrix. ZnO is known to exert antibacterial effects through a combination of reactive oxygen species (ROS) generation and release of Zn^2+^ ions. These species can damage microbial cell membranes, proteins, and DNA, leading to cell death. Notably, this mechanism—often described as a particle-mediated internalization mechanism strategy when particles are internalized by bacteria—provides broad-spectrum efficacy and makes the development of bacterial resistance less likely [47,48,49]. In the present system, ZnO is evenly dispersed in the coating, ensuring consistent antimicrobial action across the surface. The integration of antimicrobial functionality complements the barrier performance achieved at the critical film thickness, creating a packaging material that not only limits oxygen ingress but also actively suppresses microbial growth. This multifunctional behavior is particularly advantageous for dry and semi-dry food applications, where combined control of oxidation and microbial contamination is essential for maintaining product quality and safety.

### 3.4. Supporting Properties and Sustainability Considerations

Beyond barrier and antimicrobial performance, the practical applicability of paper-based packaging materials requires adequate mechanical integrity and compatibility with existing recycling infrastructures. Accordingly, key supporting properties relevant to handling, use, and end-of-life considerations were qualitatively assessed for the optimized coating system. Preliminary folding endurance tests indicated that the PVA–amylose-based coating maintained sufficient flexibility to withstand repeated bending without visible cracking or delamination. The inclusion of amylose contributed to improved film toughness and flexibility, supporting the suitability of the coating for flexible and semi-flexible packaging formats. While extensive mechanical optimization was beyond the scope of the present study, the observed performance confirms that the barrier and antimicrobial enhancements were not achieved at the expense of basic mechanical integrity.

From a sustainability perspective, the coating system is composed predominantly of water-soluble and bio-derived components, enabling compatibility with conventional paper recycling processes. Repulpability testing demonstrated a pulp recovery yield exceeding 95%, indicating that the coated paper can be effectively recycled without significant interference from the barrier layer. This performance contrasts favorably with conventional polyethylene-coated papers, which typically exhibit substantially lower fiber recovery due to persistent plastic layers.

It should be noted that, despite the demonstrated oxygen barrier performance, antimicrobial functionality, and high recyclability, achieving effective moisture barrier performance using fully water-based and bio-derived polymer coatings on paper remains inherently challenging. The hydrophilic nature of polysaccharide and PVA-based systems, while advantageous for repulpability and environmental compatibility, intrinsically limits resistance to water vapor transport. Consequently, further improvements in WVTR performance will require careful material selection and structural design strategies that balance moisture resistance with the preservation of recyclability. Collectively, these supporting results reinforce the feasibility of the proposed coating architecture as a sustainable alternative to plastic-laminated paper packaging. By integrating effective oxygen barrier performance, active antimicrobial functionality, acceptable mechanical behavior, and high recyclability within a single coating system, the present approach demonstrates a promising pathway toward next-generation, bio-based paper packaging materials. Future work will focus on comprehensive life cycle assessment and further material optimization to quantitatively address moisture barrier performance while maintaining the environmental advantages of the system.

## 4. Conclusions

This study demonstrates that high-performance functional coatings on porous paper substrates can be effectively achieved through process-controlled structural design, rather than relying on material chemistry alone. Single-layer PVA-based coatings were unable to provide meaningful oxygen barrier performance due to substrate-induced defects and pinhole formation. In contrast, a multi-layer deposition strategy enabled the formation of a continuous coating layer and identified a critical dry film thickness of approximately 5–6 μm as the threshold required for functional oxygen barrier behavior. Above this critical thickness, the PVA–amylose/ZnO coating exhibited a pronounced reduction in oxygen transmission rate, reaching ~15 cc/m^2^·day·atm at 20 °C and 65% RH. These results establish a clear process–structure–property relationship and define a practical design rule for applying water-based biopolymer coatings on porous substrates. In addition, the incorporation of ZnO nanoparticles imparted effective antimicrobial functionality, resulting in near-complete suppression of both *Escherichia coli* and *Staphylococcus aureus*, thereby enabling a multifunctional coating that combines passive barrier performance with active microbial inhibition. Importantly, the coating system maintains key sustainability advantages, including high repulpability and compatibility with conventional paper recycling processes.

Overall, this work presents a scalable and application-relevant strategy for developing multifunctional polymer films on paper, offering a promising alternative to conventional plastic-based barrier materials for sustainable food packaging applications.

## Figures and Tables

**Table 1:** Effect of coating strategy on film thickness, oxygen barrier performance, and structural integrity of PVA–amylose coatings on paper.

Number of Layers	Thickness ** (μm, range)	OTR * (cc/m^2^·day·atm)	KIT Rating	Structural Integrity
1×	0.77–1.32	>1000	1–3	(Pinhole-dominated)
2×	2.1–2.8	450–650	4–6	(Partial continuity)
4×	3.9–4.6	85–145	8–10	(Improved but defects remain)
6×	5.40–6.57	15.2–15.6	11–12	(Acceptable continuity)

Note: * OTR measured at 20 °C and 65% relative humidity. KIT rating determined according to TAPPI T559; higher values indicate improved coating continuity and resistance to liquid penetration.** Thickness values are reported as ranges (minimum–maximum) based on multiple measurements across each sample to account for spatial thickness variation.

**Table 2:** Antimicrobial activity of PVA–amylose/ZnO-coated paper against *E. coli* and *S. aureus*.

Sample	ZnO Content	Reduction (%)	Qualitative Assessment
Uncoated paper	0	~0	No activity
PVA–amylose/ZnO	0 wt%	<50	Limited
PVA–amylose/ZnO	0.5 wt%	90.00–93.78	Excellent
PVA–amylose/ZnO	1 wt%	>99.99%	Excellent

## Data Availability

The original contributions presented in this study are included in the article. Further inquiries can be directed to the corresponding author.
